# Supplementary material for: Frataxin gene editing rescues Friedreich’s ataxia pathology in dorsal root ganglia organoid-derived sensory neurons
Source: Nat Commun. 2020 Aug 21;11:4178. doi: 10.1038/s41467-020-17954-3 (PMC7442818; doi:10.1038/s41467-020-17954-3)
Supplement: Supplementary file 1 — Supplementary Information [file 41467_2020_17954_MOESM1_ESM.pdf]

SUPPLEMENTARY INFORMATION

Frataxin gene editing rescues Friedreich's ataxia pathology in dorsal root ganglia organoid-derived sensory neurons

Mazzara, Muggeo et al.

Supplementary Figure 1

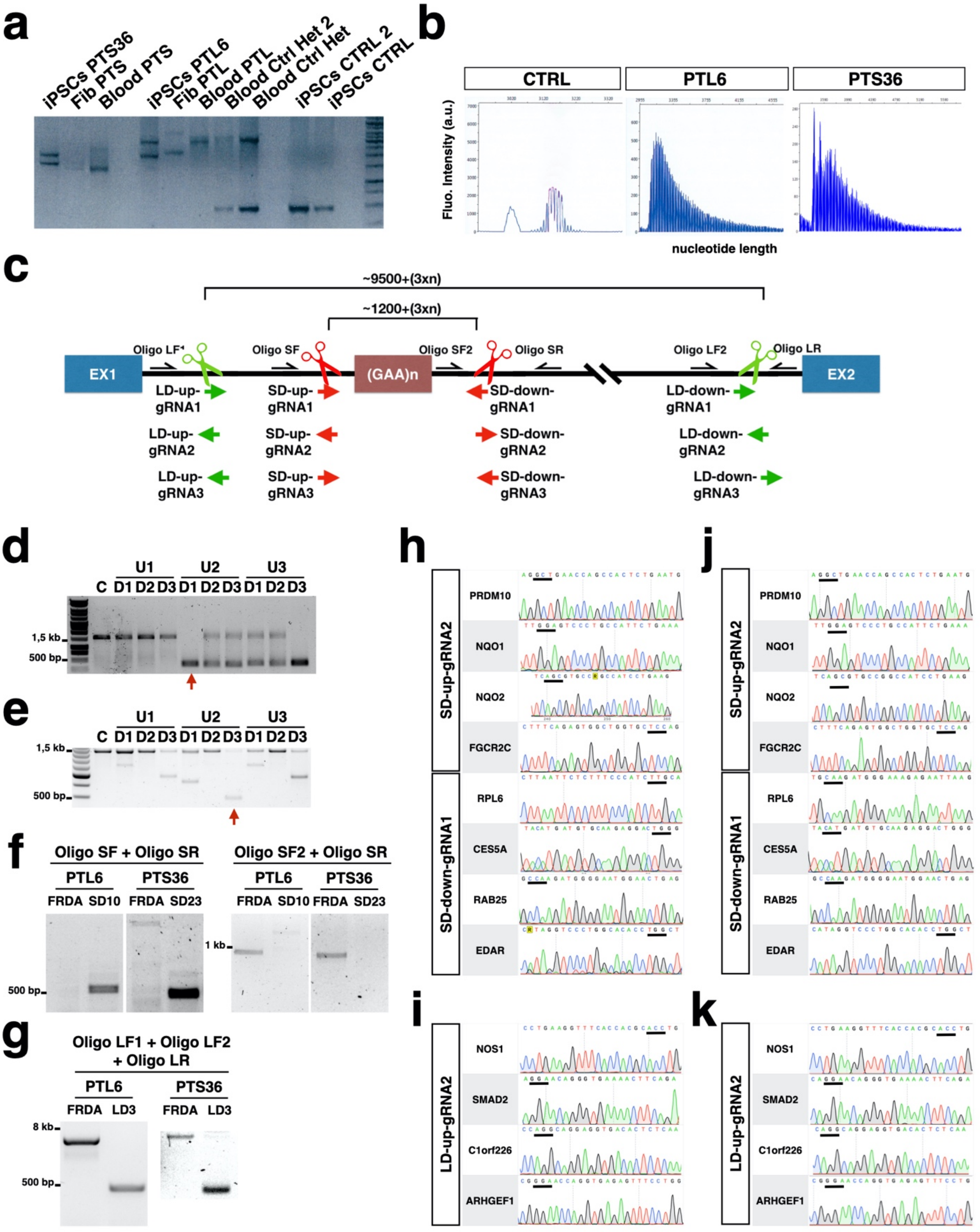

Supplementary Figure 1. Validation of short and long genomic deletions of FXN intron 1. (**a**) Genomic analysis for *FXN* intronic GAA tract on heathy controls (fibroblasts and iPSCs lines), two heterozygous controls (blood samples), PTL6 (blood samples, fibroblasts and iPSCs) and PTS36 (blood samples, fibroblasts and iPSCs). (**b**) Fluorescent repeat-primed PCR analysis of the *FXN* locus. The peak profiles of healthy control iPSCs with two alleles in the normal size range (left panel) are distinct from the profiles found in FRDA patients PTL6 (central panel), and PTS36 (right panel). (**c**) Schematic illustration of the two strategies for CRISPR/Cas9 based deletions of *FXN* intron 1, showing all the sgRNAs designed for the short (SD, red) and long deletion (LD, green) and the primers utilized for the genomic screenings. (**d** and **e**) T7 Endonuclease I (T7EI) assay in HeLa cells to identify the gRNA pairs for the short (SD) (**d**) and long (LD) deletion (**e**) with the highest INDEL rate activity (red arrows). (**f** and **g**) Genomic analysis after transfection of the sgRNA/Cas9 expressing plasmids in PTL6 and PTS36 iPSC clones to identify clones with the short (**f**) and long deletion (**g**). (**h**-**k**) Analysis of the 4 highest ranked off-target genomic sites for each sgRNA utilized for the genomic deletions (**h** and **i**, PTL-SD and PTL-LD, respectively; **j** and **k**, PTS-SD and PTS-LD, respectively). No predicted off-target sites are associated with the LD-down-sgRNA3. In the chromatograms, PAM sequences are underlined. Note the single nucleotide polymorphisms (SNPs) are indicated in yellow.

Supplementary Figure 2

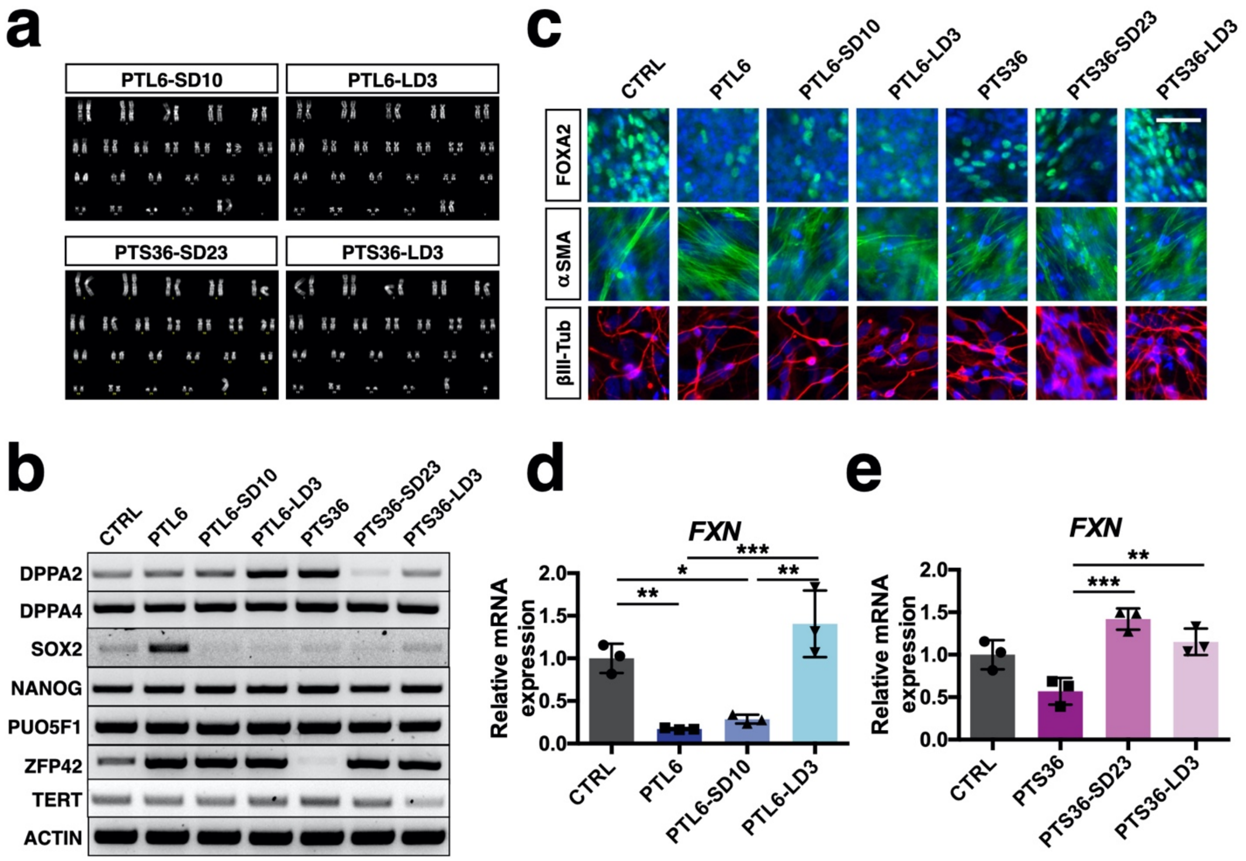

Supplementary Figure 2. Validation of pluripotency in PTL6 and PTS36 patient and their targeted iPSC lines. (**a**) Normal karyotype of the targeted PTL6 and PTS36 iPSC lines. (**b**) Expression of crucial pluripotency markers by RT–PCR in control (CTRL) and untreated and targeted iPSCs. (**c**) iPSC differentiated cultures show a mix of cells of all three germinal layers including endoderm (FoxA2), mesoderm (□SMA) and ectoderm (ßIII-Tubulin). Scale bar, 10µm. (**d** and **e**) Quantitative analysis of *FXN* transcript levels in PTL6 (**d**), PTS36 (**e**) and their targeted iPSC lines. Expression levels are normalized to actin. Mean ± s.d., n = 3 independent experiments, 8-12 organoids/line/experiment. * P<0.05; ** P<0.01; *** P<0.001; one-way ANOVA with Bonferroni correction.

Supplementary Figure 3

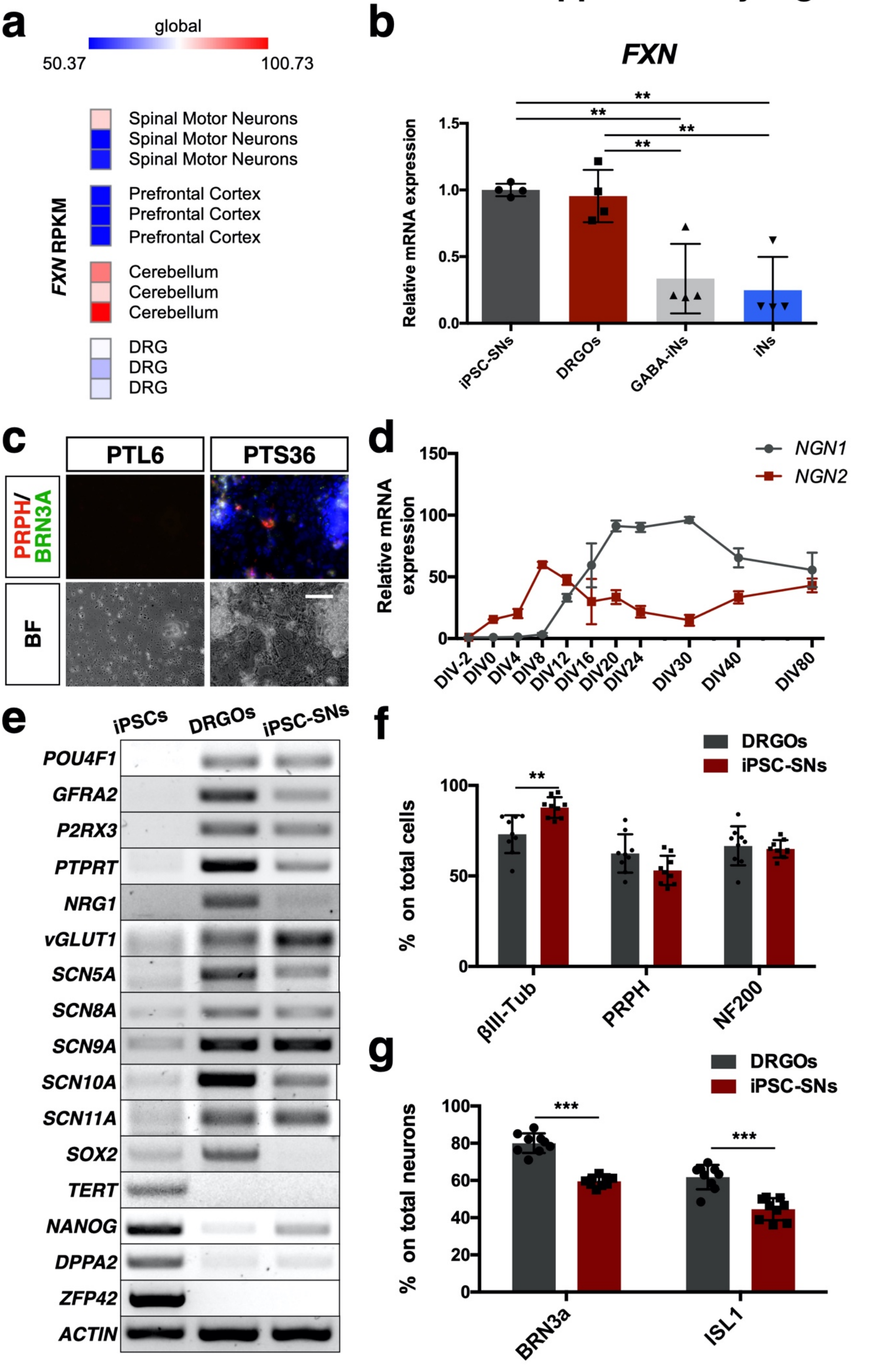

Supplementary Figure 3. *FXN* gene expression in different neuronal subtypes and characterization of iPSC-derived DRGOs. (**a**) Heatmap showing absolute *FXN* gene expression levels (RPKM) in different human neuronal subtypes or CNS structures. (**b**) *FXN* transcriptional levels in different iPSCs-derived neuronal subtypes including GABAergic (GABA-iNs) and cortical excitatory (iNs) neurons. Expression levels are normalized to actin. Mean ± s.d., n = 4 independent experiments, 10-12 organoids or pooled 2D-differentiated peripheral (iPSC-SNs) and GABA neurons/experiment. ** P<0.001; one-way ANOVA with Bonferroni correction. (**c**) Immunocytochemistry and BF images acquired in FRDA patients 2D-differentiated peripheral neurons (iPSC-SNs) at DIV 40 for neuronal projections marker PRPH and the transcription factors BRN3A. n = 3 independent experiments. Scale bar, 50μm. (**d**) Quantitative analysis of *NEUROG1* and *NEUROG2* transcript levels during DRGO differentiation. Expression levels are normalized to Actin. Mean ± s.d., n = 3 independent experiments, 8-12 organoids/line/experiment. (**e**) Transcriptional expression analysis of sensory neuron markers in iPSCs, DRGOs and iPSC-SNs at DIV 40. n = 3 independent experiments, 10 organoids or pooled iPSCs and PerNs. (**f** and **g**) Quantifications of immunocytochemistry in DRGOs (**f**) and 2D-differentiated peripheral neurons (iPSC-SNs) (**g**) at DIV 40. n=9 independent experiments, 3 organoids or pooled iPSCs and iPSC-SNs/experiment. ** P<0.01; *** P<0.001; Two-way ANOVA.

Supplementary Figure 4

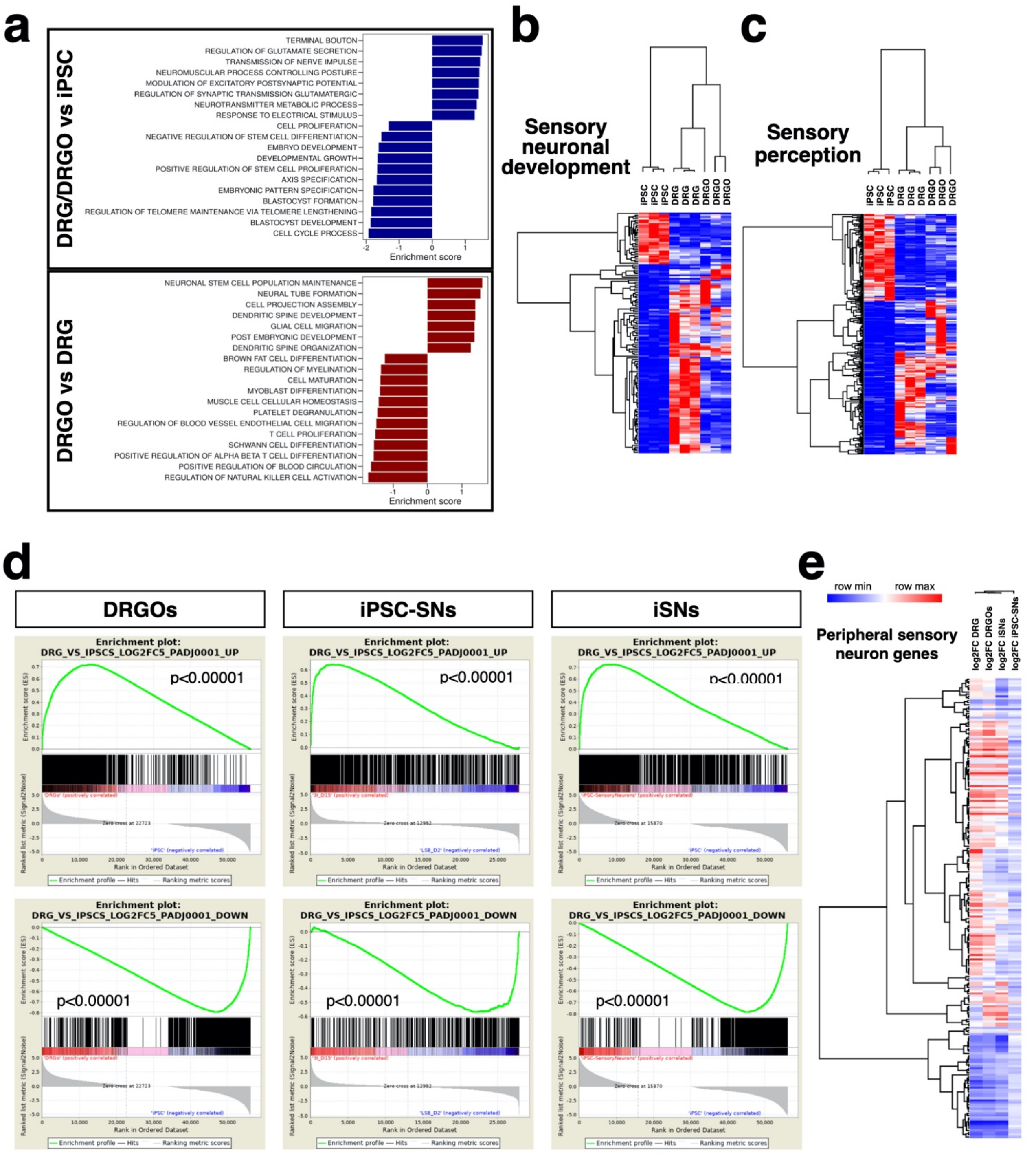

Supplementary Figure 4. Transcriptome comparative analysis of different peripheral sensory neurons models. (**a**) Histograms displaying representative functionally enriched gene ontology (GO) categories in DRGs/DRGOs versus iPSCs (**top**), and DRGOs versus DRGs (**bottom**). (**b** and **c**) Gene expression heatmaps showing the differentially expressed genes belonging to aggregated GO

categories associated with sensory organ development (**b**) and sensory perception (**c**); the correlation between samples is also shown as an unsupervised hierarchical clustered dendrogram on the side. (**d**) Gene Set Enrichment Analysis (GSEA) enrichment plots relative to up- and down-regulated genes in primary human DRG versus iPSC, in the three different approaches to generate sensory neurons, namely: DRGOs, iPSC-derived (iPSC-SNs) and induced sensory neurons (iSNs). (**e**) Heatmap showing log_2 Fold Changes (log_2FC) of aggregated peripheral sensory neuron GO categories in human somatic DRGs, DRGOs, iSNs and iPSC-SNs.

Supplementary Figure 5

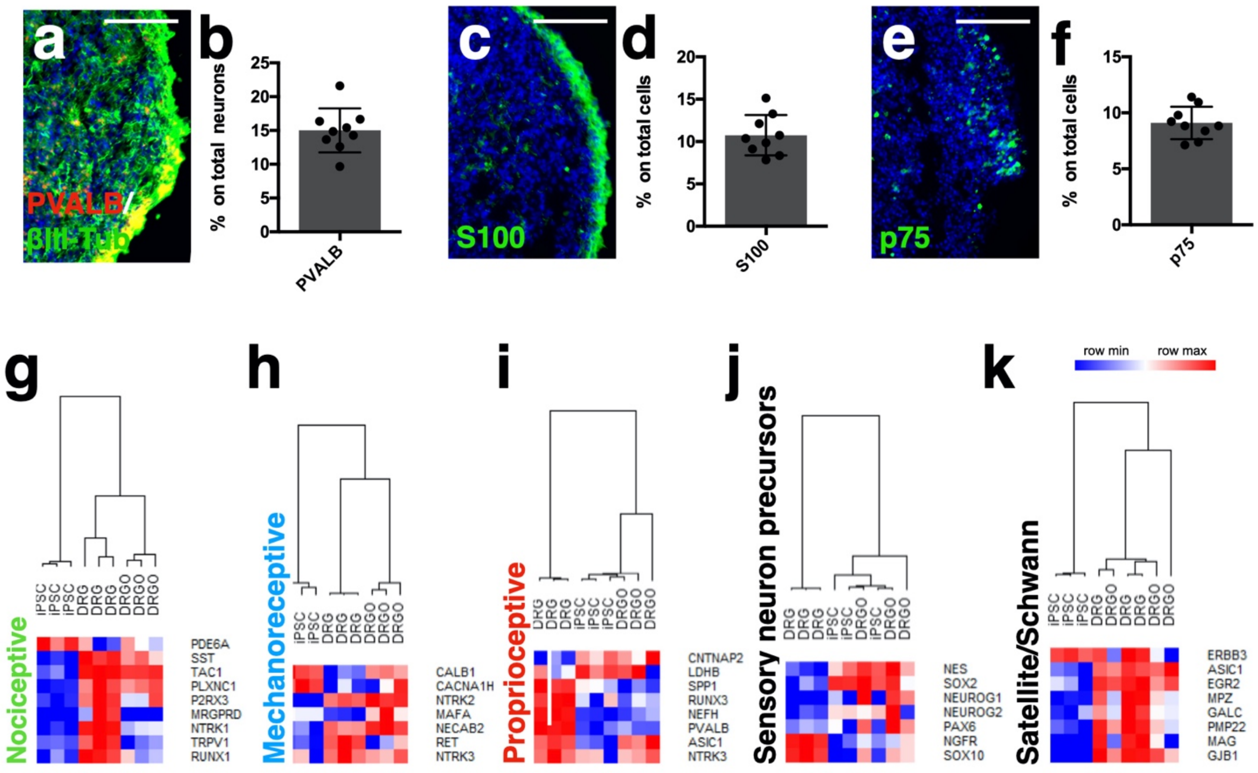

Supplementary Figure 5. Cellular subtype specification in DRGOs. (**a-f**) Immunocytochemistry and quantification of DIV 40 DRGO cellular subtypes, including PVALB+ sensory neurons (**a** and **b**), S100+ satellite-like cells (**c** and **d**) and p75+ precursor (**e** and **f**). Mean ± s.d. n = 9 independent experiments, 3 organoids/experiment. Scale bars, 100µm. (**g-k**) DIV 40 DRGO gene expression heatmaps showing the differentially expressed genes belonging to aggregated GO categories associated with nociceptive (**g**), mechanoreceptive (**h**), proprioceptive (**i**) neurons, sensory neuron precursors (**j**); satellite/Schwann cells (**k**); the correlation between samples is also shown as an unsupervised hierarchical clustered dendrogram.

Supplementary Figure 6

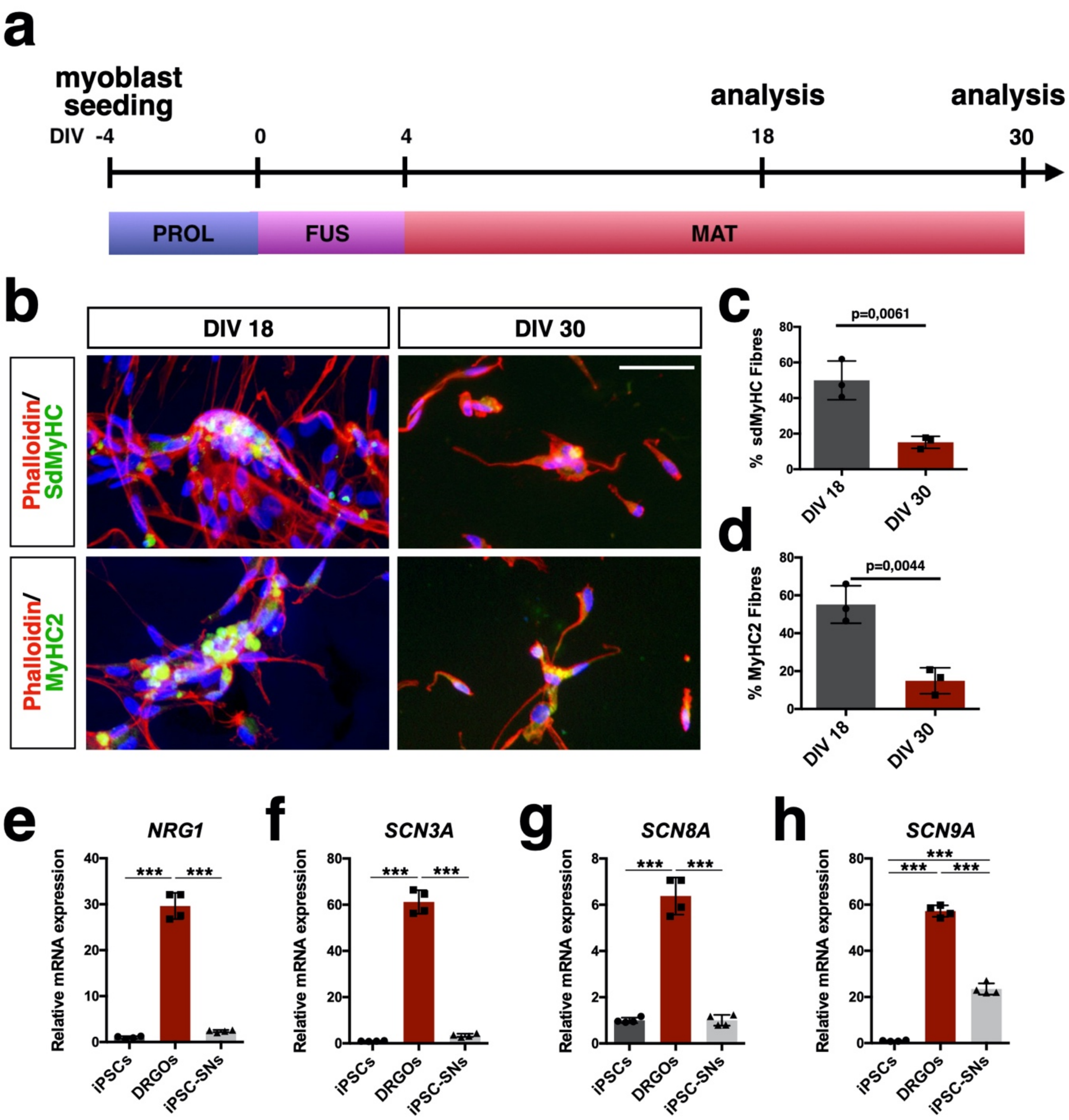

Supplementary Figure 6. *In vitro* differentiation of human intrafusal muscle fibers. (**a**) Schematic representation of differentiation of primary human myoblasts into intrafusal muscle fibers. PROL, proliferation medium; FUS, fusion medium; MAT, maturation medium. (**b**) Representative immunofluorescence images showing intrafusal muscle fibers labeled by perinuclear staining of the slow-tonic myosin heavy chain (S46) and fast myosin 2 heavy chain (A4.74) that preferentially mark respectively bag and chain fibers at DIV 18 and DIV 30. Scale bar, 50µm. (**c** and **d**) Quantification of multinucleated fibers expressing the intrafusal fiber markers S46 (**c**) and A4.74 (**d**). Mean ± s.d., n = 3 independent experiments, 3 coverslips/condition/experiment. Two-sided Student's T-test. (**e-h**)

Quantitative PCR (qPCR) analysis showing relative expression levels in DRGOs or 2D-differentiated peripheral neurons (iPSC-SNs) respect to iPSCs expression levels for the genes encoding for NRG1 (**e**) and the sodium channels Nav1.3 (SCN3A) (**f**), Nav1.6 (SCN8A) (**g**) and Nav1.7 (SCN9A) (**h**). Expression levels are normalized to actin. Mean ± s.d., n = 4 independent experiments, 10 organoids or pooled iPSCs and iPSC-SNs/experiments. *** $P<0.001$; one-way ANOVA with Bonferroni correction.

Supplementary Figure 7

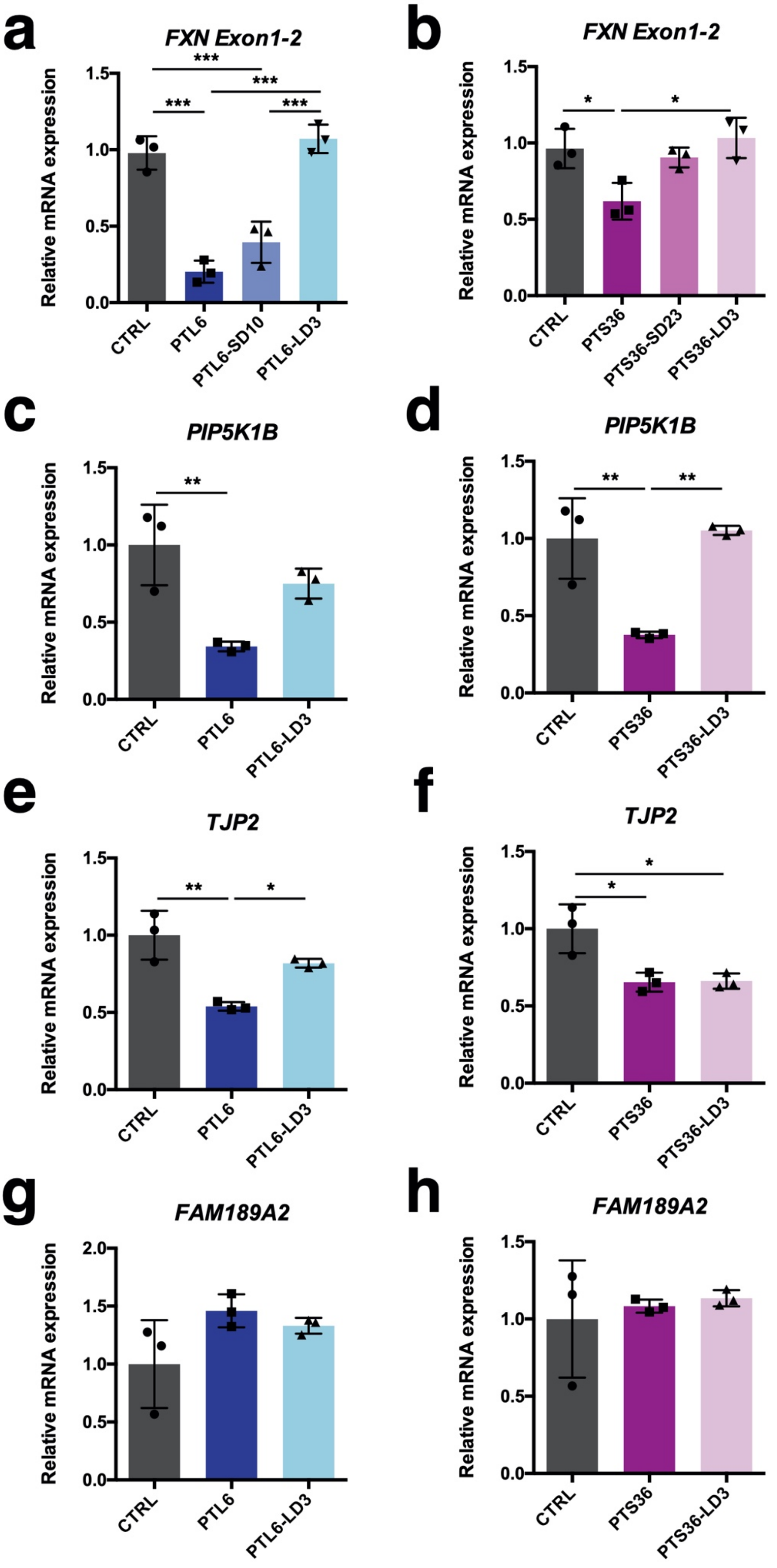

Supplementary Figure 7. Frataxin flanking genes expression in untreated and LD patients iPSC-derived DRGOs. (**a** and **b**) Quantitative analysis in control, FRDA patient and isogenic DRGOs of *FXN* exons 1 and 2 junction. Mean ± s.d., n = 3 independent experiments, 8-12 organoids/line/experiment. * P<0.05; *** P<0.001, one-way ANOVA with Bonferroni correction. (**c**-**h**) Quantitative analysis in control, FRDA patients and LD isogenic DRGOs of FXN flanking genes *PIP5K1B* (**c** and **d**), *TJP2* (**e** and **f**) and *FAM189A2* (**g** and **h**) transcript levels. Expression levels are normalized to actin. Mean ± s.d., n = 3 independent experiments, 8-12 organoids/line/experiment. * P<0.05; ** P<0.01; *** P<0.001, one-way ANOVA with Bonferroni correction.

Supplementary Figure 8

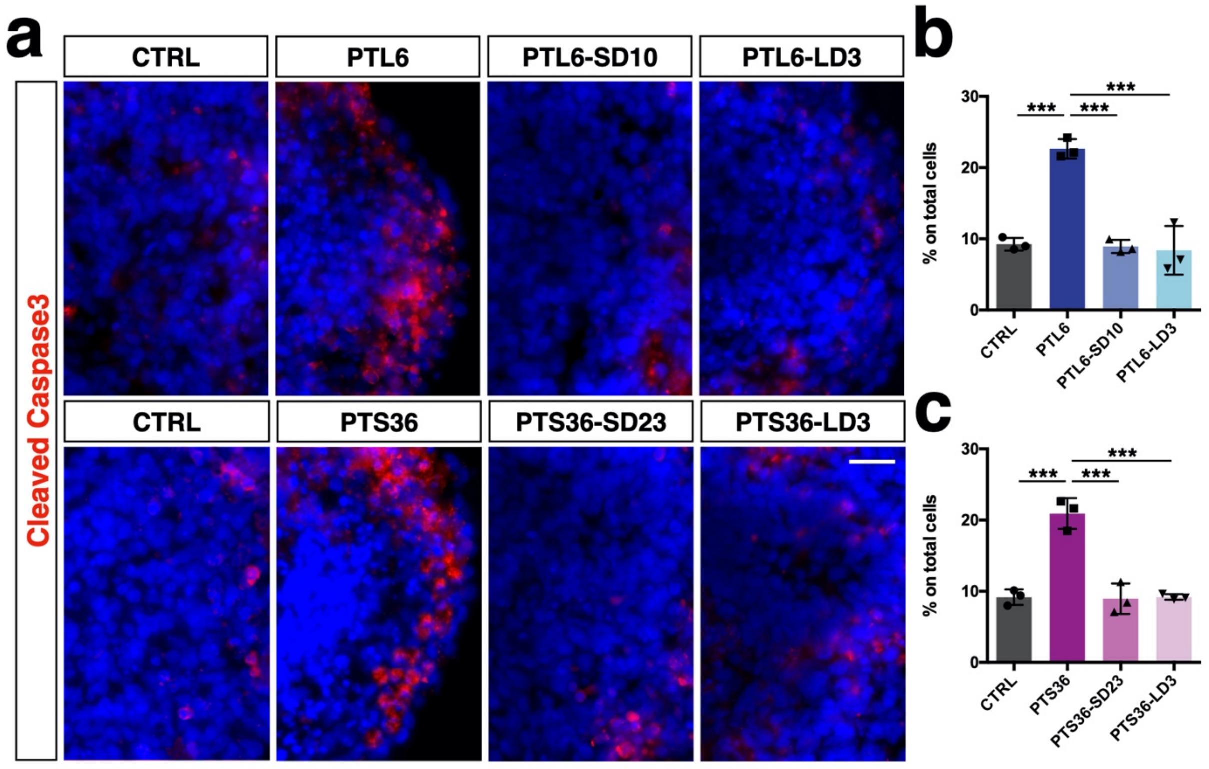

Supplementary Figure 8. Rescue of cell apoptosis in CRISPR/Cas9 edited FRDA-LD DRGOs. (**a-c**) Representative immunofluorescence images of cryosectioned DIV 16 FRDA patients line derived DRGOs along with short and long deletion isogenic lines, respectively, as compared with a control (CTRL) healthy donor derived DRGO stained for the apoptotic marker cleaved Caspase 3 (**a**, red) and quantification of positive cells in PTL6 and its isogenic lines (**b**) and PTS36 and its isogenic lines (**c**). Mean ± s.d., n = 3 independent experiments, 3-6 organoids/line/experiment. *** P<0.001; one-way ANOVA with Bonferroni correction. Scale bar, 10μm.

Supplementary Figure 9

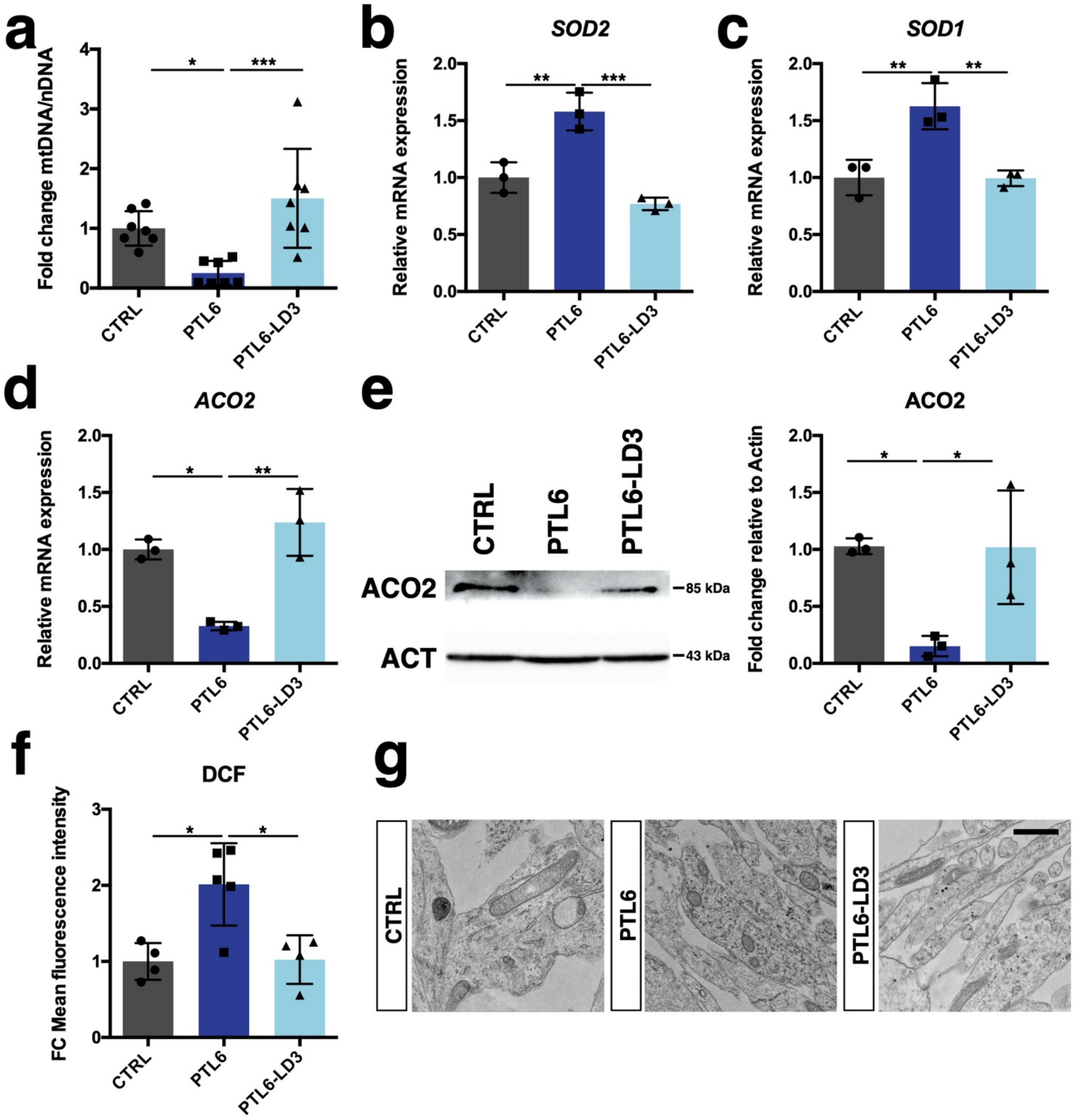

Supplementary Figure 9. Mitochondrial biogenesis, Aconitase 2 protein levels and SOD1/2 gene expression in PTL6 and PTL-DL3 DRGOs. (**a**) Quantitative analysis of the mitochondrial DNA (mtDNA) copies normalized on genomic nuclear DNA (nDNA) in PTL6 and PTL6-DL3 DRGOs. Mean ± s.d., n = 7 independent experiments, 8-12 organoids/line/experiment. * $P<0.05$; *** $P<0.001$; one-way ANOVA with Bonferroni correction. (**b**-**d**) Quantitative analysis of mitochondrial *SOD2* (**b**), cytosolic *SOD1* (**c**) and Aconitase 2 (*ACO2,* **d**) transcript levels in control, PTL6 and PTL6-LD3 DRGOs. Expression levels are normalized to Actin. Mean ± s.d., n = 3 independent experiments, 8-

12 organoids/line/experiment. * $P<0.05$; ** $P<0.01$; *** $P<0.001$; one-way ANOVA with Bonferroni correction. (**e**) Immunoblot and relative quantification of Aconitase 2 (ACO2) protein levels in control, PTL6, and PTL6-LD3 DRGOs. Protein levels are normalized to actin. Mean ± s.d., n = 3 independent experiments 24-36 organoids/line/experiment. * $P<0.05$; one-way ANOVA with Bonferroni correction. (**f**) H2DCFA signal was estimated by FACS analysis in control, PTL6 and PTL6-LD3 DRGOs and plotted as Mean Fluorescence Intensity (MFI) fold change. Mean ± s.d., n = 4 independent experiments, 3 organoids/line/experiment. * $P<0.05$; one-way ANOVA with Bonferroni correction. (**g**) Original electronic microscopy images of mitochondria along DRGO axons showed in Figure 7D. Scale bar: 1μm.

Supplementary Table 1: sgRNA sequences

Name	Sequence
SD-up-gRNA1-Fw	CTGGTACGCCGCATGTATTAGGG
SD-up-gRNA2-Rev	CCCTTCAGAGTGGCTGGTACGCC
SD-up-gRNA3-Fw	CCCTTCAGAGTGGCTGGTACGCC
SD-down-gRNA1-Rev	CCATAGTTCCCTTGCACATCTTG
SD-down-gRNA2-Fw	ATAGTTCCCTTGCACATCTTGGG
SD-down-gRNA3-Rev	CCCTTGCACATCTTGGGTATTTG
LD-up-gRNA1-Fw	TGACGCCCATTTTGCGGACCTGG
LD-up-gRNA2-Rev	CCTGGAACGAGGTGAAACTTTCA
LD-up-gRNA3-Rev	CCCATTTTGCGGACCTGGTGTGA
LD-down-gRNA1-Fw	CCGAAACTAGCTTGGGTGAGGGG
LD-down-gRNA2-Rev	CCTCTGCTAGTTCCGTGCATACT
LD-down-gRNA3-Fw	TAGTTCCGTGCATACTCTACAGG

Supplementary Table 2: List of primers

Name	Sequence
ACO2 Fw	TTGAGCCCAACGAGTACATCC
ACO2 Rev	GTCCATACACAATCTTCTCCGAG
ACTIN Fw	ACCCCAGCCATGTACGTT
ACTIN Rev	GGTGAGGATCTTCATGAGGTAG
ARHGEF1 Fw	GCGGTTGTGGATTTGAGTTA
ARHGEF1 Rev	CTCAGGGAAGGTTCTGGAAA
B2M (DNA) Fw	TGCTGTCTCCATGTTTGATGTATCT
B2M (DNA) Rev	TCTCTGCTCCCCACCTCTAAGT
CACNA1H Fw	ATGCTGGTAATCATGCTCAACTG
CACNA1H Rev	AAAAGGCGAAAATGAAGGCGT
CES5A Fw	CTGGCAACGGTAGAGGAAAG
CES5A Rev	CGCATGGAGGAAAGGAGATA
CLORF226 Fw	CCATCACATTTCTCCGACTG
CLORF226 Rev	TAATGGCAATGGAGGACAGA
DPPA2 Fw	GATGCAAAATACCAGCCCTTCCC
DPPA2 Rev	CGTTTCCTCGAACATCGCTGTAA
DPPA4 Fw	GACCTCCACAGAGAAGTCGAG
DPPA4 Rev	AGGTGGCAGTTTAGAAGGTAATG
EDAR Fw	TGGCACACCTCTCTGACACT
EDAR Rev	AAGGAAGAGCAGTCCTGGTG
FAM189A2 Fw	TGCCTTGAAACTCTTTCCGGT
FAM189A2 Rev	TGGTTGCGAATATCTGGAGGT
FCGR2C Fw	TGGGAGAAGGAGGAGATGAG
FCGR2C Rev	AGCCTATGTTTCCTGTGCAGT
FXN Fw	GGAAACGCTGGACTCTTTAGC
FXN Rev	CCAGTTTGACAGTTAAGACACCA
FXN EXON1-2 Fw	CACCGACATCGATGCGACC

FXN EXON1-2 Rev	GGCCCAAAGTTCCAGATTTCC
FXN LD Fw 1	TCCATGCTTGTCACTTCTCTG
FXN LD Fw 2	GGATTCTCGAAACTGGTGGT
FXN LD Rev	ACTGGGAGGGCATACTGAAA
FXN SD Fw1	GTGGCGTAACTGGGTCAGAT
FXN SD Fw2	ATAGCCCTTAACAGCCACCA
FXN SD Rev	TCCCCACCCTTTTCTC
FXN GAA exp Fw	GGGATTGGTTGCCAGTGCTTAAAAGTTAG
FXN GAA exp Rev	GATCTAAGGACCATCATGGCCACACTTGCC
GFRA2 Fw	GGGCTCTTATGCTGGCATGAT
GFRA2 Rev	AGTCCCTGAGGAACTTCTCAC
mt-TL1 (DNA) Fw	CACCCAAGAACAGGGTTTGT
mt-TL1 (DNA) Rev	TGGCCATGGGTATGTTGTTA
NANOG Fw	CCTCCATGGATCTGCTTATT
NANOG Rev	ATCTGCTGGAGGCTGAGGTA
NGN1 Fw	GCTCTCTGACCCCAGTAGC
NGN1 Rev	GCGTTGTGTGGAGCAAGTC
NGN2 Fw	AGGAAGAGGACGTGTTAGTGC
NGN2 Rev	GCAATCGTGTACCAGACCCAG
NOS1 Fw	CTGGGATTTCTGGTGAAGGA
NOS1 Rev	AGGCTGACTCTGGTTGCTTT
NQO1 Fw	AGCAACAGCATGAAGCAAAC
NQO1 Rev	TCAGAACCATCCACCTACCC
NQO2 Fw	AGACTGCTGCTCCAAAGCTG
NQO2 Rev	CCCTCCCTCCAACTATCCAT
NRG1 Fw	AGTCCTTCGGTGTGAAACCAG
NRG1 Rev	TGCGAAGTTCTGACTTCCCTG
NTRK1 Fw	AACCTCACCATCGTGAAGAGT
NTRK1 Rev	TGAAGGAGAGATTCAGGCGAC
P2RX3 Fw	TCCCCAGGCTACAACTTCAG
P2RX3 Rev	TGTTGAACTTGCCAGCATTC
PIP5K1B Fw	TGACCCCAGCACATCACTAC
PIP5K1B Rev	GCTCCAGGGTTAGACAGTTCT
POU4F1 Fw	CGTACCACACGATGAACAGC
POU4F1 Rev	AGGAGATGTGGTCCAGCAGA
POU5F1 Fw	AGAAAGCGAACCAGTATCGAGAA
POU5F1 Rev	CTCAAAATCCTCTCGTTGTGCAT
PRDM10 Fw	TGTGGAACCTCTCCTGCTTT
PRDM10 Rev	CTGGTGGTGGGAGGAAGAT
PTPRT Fw	CATACCTGTGGATCAAGCCAAA
PTPRT Rev	TCTCATACTCAACATCGGGGTC
PVALB Fw	AAGAGTGCGGATGATGTGAAG
PVALB Rev	GCCTTTTAGGATGAATCCCAGC
RAB25 Fw	CCTGCCTAACCTTGAGTTGG
RAB25 Rev	ACACCAGGGATTTGGGTTTC
RUNX1 Fw	TGAGCTGAGAAATGCTACCGC

RUNX1 Rev	ACTTCGACCGACAAACCTGAG
RUNX3 Fw	AGGCAATGACGAGAACTACTCC
RUNX3 Rev	CGAAGGTCGTTGAACCTGG
SCN3A Fw	TGTGTCCCCTACCTTGTTCC
SCN3A Rev	CATGACCAGGAAGAGCAGGA
SCN5A Fw	CCGCCATTTACACCTTTGAGT
SCN5A Rev	CGCTGAGGCAGAAGACTGTG
SCN8A Fw	ACAATGTTGGGGCAGGATAC
SCN8A rev	GGTGAAGAAGGAGCCGAAG
SCN9A Fw	ACCTATCTCTGCTTCAAGTTGC
SCN9A Rev	TGGGCTGCTTGTCTACATTAAC
SCN10A Fw	CTGTCGATGTCTCGGCATTC
SCN10A Rev	TGGGCACTTCTGTTCAGACTC
SCN11A Fw	GAAATGCTTACCTCGCTCTG
SCN11A Rev	GCTCTCAAACTCTGGCTGTTG
SMAD2 Fw	CATGGTTGAGGACCAATTCA
SMAD2 Rev	CCTTAGACTGTGGGATGCAA
SOD1 Fw	GGTGGGCCAAAGGATGAAGAG
SOD1 Rev	CCACAAGCCAAACGACTTCC
SOD2 Fw	GCTCCGGTTTTGGGGTATCTG
SOD2 Rev	GCGTTGATGTGAGGTTCCAG
SOX2 Fw	CAAGCTCCTTCAACTGGTTCTGT
SOX2 Rev	CTTAGAATGATGCAAGCCAGGTC
TERT Fw	ACCAAGAAGTTCATCTCCCTGGG
TERT Rev	AAAGAAAGACCTGAGCAGCTCGA
TFAM Fw	GTGATTCACCGCAGGAAAAGC
TFAM Rev	GTGCGACGTAGAAGATCCTTTC
TJP2 Fw	GGGAAGGTCGCTGCTATTGT
TJP2 Rev	CTCTCGCTGTAGCCACTCC
TRPV1 Fw	GCACAGGAGAGCAAGAACATC
TRPV1 Rev	GTCCAGTTCACCTCGTCCAC
vGLUT1 Fw	AAGCTAGCGGGTCGTGCT
vGLUT1 Fw	ACTCAGCTCCAGCGTC
ZFP42 Fw	CAGATCCTAAACAGCTCGCAGAAT
ZFP42 Rev	GCGTACGCAAATTAAAGTCCAGA

Supplementary Table 3: Primers for the epigenetic analysis

Name	Sequence
EX1 Fw	AGTGCTAAGCTGGGAAGTTCT
EX1 Rev	CCCGAGAGTCCACATGCTG
NC5' Fw	CGGTTGCATTTACACTGGCT
NC5' Rev	CGCAGAGAAGTGACAAGCAT
UpSD Fw	TAACCTCTCTGAGACGTGGC
UpSD Rev	GGTTTCCTCCTTTCAAGCCG
DwSD Fw	TAAGGGGCTGAGTGTGCTAC

DwSD Rev	TGGCAGTATTGAGAGGTGGG
UC3’ Fw	ATGGGTTTGCTTTGGTCTGC
UC3’ Rev	TTCTCCTCTCCCCTGGTTCC
Prom Fw	5’-ACTTGGAAGGCTGAAATGGG-3’
Prom Rev	5’-CCAGGATGGTCTCGATCTAC-3’

Supplementary Table 4: List of antibodies

Antigen	Distributor
Anti-mouse-647	Thermo A21463
Anti-mouse-488	Thermo A21202
Anti-mouse-594	Thermo A21203
Anti-rabbit-488	Thermo A21206
Anti-rabbit-546	Thermo 10040
Anti-chicken-647	Thermo A21449
Anti-guinea Pig-488	Thermo A11073
Anti-goat-488	Thermo A32814
Anti-Mouse Immunoglobulins/HRP	DAKO P0447
Anti-Rabbit Immunoglobulins/HRP	DAKO P0448
A4.47-s	DSHB AB528383
ACO2	Antibody Verify AAS01563C
ACTIN	Merck A3853
BRN3a	Milipore MAB1585
CALRETININ	Abcam ab702
FXN	Abcam 110328
vGlut1	Abcam 77822
vGlut1	Synaptic Systems 135011
ISLET1	Hybrid Bank 39.4D
NF200	Abcam 4680
P75 (CD271)	Promega G3231
PVALB	Merck P3088
PRPH	Millipore AB1530
S46-s	DSHB AB528376
S100	Abcam ab868
TOMM20	Novus NBP1-81556
TRKA	R&D Systems AF175
TRKB	R&D Systems MAB3971-100
TRKC	Abcam ab43078
Tuj1	Covance PRB-435P
