# Supplementary material for: Frataxin gene editing rescues Friedreich’s ataxia pathology in dorsal root ganglia organoid-derived sensory neurons
Source: Nat Commun. 2020 Aug 21;11:4178. doi: 10.1038/s41467-020-17954-3 (PMC7442818; doi:10.1038/s41467-020-17954-3)
Supplement: Supplementary file 3 — Description of Additional Supplementary Files [file 41467_2020_17954_MOESM3_ESM.pdf]

Description of Additional Supplementary Files

File Name: Supplementary Movie 1
Description: In vitro reconstitution of the muscle spindle.
