# Supplementary material for: Frataxin gene editing rescues Friedreich’s ataxia pathology in dorsal root ganglia organoid-derived sensory neurons
Source: Nat Commun. 2020 Aug 21;11:4178. doi: 10.1038/s41467-020-17954-3 (PMC7442818; doi:10.1038/s41467-020-17954-3)
Supplement: Supplementary file 5 — Reporting Summary [file 41467_2020_17954_MOESM5_ESM.pdf]

nature research

Corresponding author(s): Vania Broccoli

Last updated by author(s): Jul 14, 2020

Reporting Summary

Nature Research wishes to improve the reproducibility of the work that we publish. This form provides structure for consistency and transparency in reporting. For further information on Nature Research policies, see Authors & Referees and the Editorial Policy Checklist .

Statistics

For all statistical analyses, confirm that the following items are present in the figure legend, table legend, main text, or Methods section.

n/a	Confirmed	
☐	☒	The exact sample size (*n*) for each experimental group/condition, given as a discrete number and unit of measurement
☐	☒	A statement on whether measurements were taken from distinct samples or whether the same sample was measured repeatedly
☐	☒	The statistical test(s) used AND whether they are one- or two-sided<br>*Only common tests should be described solely by name; describe more complex techniques in the Methods section.*
☒	☐	A description of all covariates tested
☒	☐	A description of any assumptions or corrections, such as tests of normality and adjustment for multiple comparisons
☒	☐	A full description of the statistical parameters including central tendency (e.g. means) or other basic estimates (e.g. regression coefficient) AND variation (e.g. standard deviation) or associated estimates of uncertainty (e.g. confidence intervals)
☒	☐	For null hypothesis testing, the test statistic (e.g. *F*, *t*, *r*) with confidence intervals, effect sizes, degrees of freedom and *P* value noted<br>*Give P values as exact values whenever suitable.*
☒	☐	For Bayesian analysis, information on the choice of priors and Markov chain Monte Carlo settings
☒	☐	For hierarchical and complex designs, identification of the appropriate level for tests and full reporting of outcomes
☐	☒	Estimates of effect sizes (e.g. Cohen's *d*, Pearson's *r*), indicating how they were calculated

Our web collection on statistics for biologists contains articles on many of the points above.

Software and code

Policy information about availability of computer code

Data collection	Acquisition images with Leica microscope.<br>Processing images with ImageJ
Data analysis	All statistical analysis was carried out in GraphPad Prism 8.0, using one-way ANOVA, two-way ANOVA, Mantel-Cox test (survival curves) and non-parametric Mann-Whitney U test (two-tailed) where unpaired t-test was applied. P- values below 0.05 were considered significant. In multi-group comparisons, multiple testing correction for pairwise tests among groups was applied using Tukey's post hoc analysis.

For manuscripts utilizing custom algorithms or software that are central to the research but not yet described in published literature, software must be made available to editors/reviewers. We strongly encourage code deposition in a community repository (e.g. GitHub). See the Nature Research guidelines for submitting code & software for further information.

Data

Policy information about availability of data

All manuscripts must include a data availability statement. This statement should provide the following information, where applicable:

- Accession codes, unique identifiers, or web links for publicly available datasets
- A list of figures that have associated raw data
- A description of any restrictions on data availability

RNA sequencing data referred to Figures 2D-F, S4A-F and S5G-K were deposited in the NCBI Gene Expression Omnibus repository with the GSE133755 GEO ID.
RNA sequencing data referred to Figures 3C-J were deposited in

Field-specific reporting

Please select the one below that is the best fit for your research. If you are not sure, read the appropriate sections before making your selection.

- [x] Life sciences
- [ ] Behavioural & social sciences
- [ ] Ecological, evolutionary & environmental sciences

For a reference copy of the document with all sections, see nature.com/documents/nr-reporting-summary-flat.pdf

Life sciences study design

All studies must disclose on these points even when the disclosure is negative.

Sample size	In vitro experiments were performed one time including at least 3 biological replicates or 2-3 times including more than 3 biological replicates.
Data exclusions	We considered random noise outliers below 5th or above 95th percentile.
Replication	At least 3 biological replicates for each single value have been obtained. Number of biological replicates are specified in the figure legends.
Randomization	Cell countings were performed on random miscroscope view fields (10x or 20x) for each sample.
Blinding	The investigators who performed the quantification of the immunostainings were blinded to the genotype of the samples

Reporting for specific materials, systems and methods

We require information from authors about some types of materials, experimental systems and methods used in many studies. Here, indicate whether each material, system or method listed is relevant to your study. If you are not sure if a list item applies to your research, read the appropriate section before selecting a response.

Materials & experimental systems

n/a	Involved in the study	
[ ]	[x]	Antibodies
[ ]	[x]	Eukaryotic cell lines
[x]	[ ]	Palaeontology
[x]	[ ]	Animals and other organisms
[x]	[ ]	Human research participants
[ ]	[x]	Clinical data

Methods

n/a	Involved in the study	
[x]	[ ]	ChIP-seq
[x]	[ ]	Flow cytometry
[x]	[ ]	MRI-based neuroimaging

Antibodies

Antibodies used

Anti-mouse-647 Thermo A21463
Anti-mouse-488 Thermo A21202
Anti-mouse-594 Thermo A21203
Anti-rabbit-488 Thermo A21206
Anti-rabbit-546 Thermo 10040
Anti-chicken-647 Thermo A21449
Anti-guinea Pig-488 Thermo A11073
Anti-goat-488 Thermo A32814
Anti-Mouse Immunoglobulins/HRP DAKO P0447
Anti-Rabbit Immunoglobulins/HRP DAKO P0448
Anti-A4.47-s DSHB AB528383
Anti-ACO2 Antibody Verify AAS01563C
Anti-ACTIN Merck A3853
Anti-BRN3a Milipore MAB1585
Anti-CALRETININ Abcam ab702
Anti-FXN Abcam 110328
Anti-vGlut1 Abcam 77822
Anti-vGlut1 Synaptic Systems 135011
Anti-ISLET1 Hybrid Bank 39.4D
Anti-NF200 Abcam 4680
P75 (CD271) Promega G3231
Anti-PV Merck P3088

Anti-PRPH Millipore AB1530
Anti-S46-s DSHB AB528376
Anti-S100 Abcam ab868
Anti-TOMM20 Novus NBP1-81556
Anti-TRKA R&D Systems AF175
Anti-TRKB R&D Systems MAB3971-100
Anti-TRKC Abcam ab43078
Anti-Tuj1 Covance PRB-435P

Validation

Anti-A4.47-s and Anti-S46-s were validated on primary human Myoblasts

Anti-ACO2, Anti-BRN3a, Anti-CALRETININ, Anti-FXN, Anti-vGlut1 (Abcam 77822 and Synaptic Systems 135011), Anti-ISLET1, Anti-NF200, Anti-PV, Anti-PRPH, Anti-TOMM20, Anti-TRKA, Anti-TRKB, Anti-TRKC and Anti-TuJ1 were validated on primary mouse DRGs and hiPSC derived Sensory neurons

Anti-S100 and P75 (CD271) were validated on primary mouse sciatic nerves and human fibroblast derived iSchwann cells

Eukaryotic cell lines

Policy information about cell lines

Cell line source(s)

Healthy control human fibroblasts DIGI were obtained from the IRCCS Carlo Besta
Healthy control human fibroblasts Neof2 were obtained from ATCC
FRDA patient fibroblasts (PTS and PTL) where obtained from the Franco Taroni's lab at the IRCCS Carlo Besta Neurological Institute
Human primary myoblasts (#48046 and #105809) were obtained from Telethon Network of Genetic Biobanks

Authentication

Healthy control human fibroblasts DIGI, Healthy control human fibroblasts Neof2 and FRDA patient fibroblasts (PTS and PTL) were authenticated by Immunofluorescence against PDGFR and Fibroblast activating protein
Human primary myoblasts (#48046 and #105809) were authenticated by Immunofluorescence against myosin heavy chain (MHC)

Mycoplasma contamination

tested negative by pcr method

Commonly misidentified lines
(See ICLAC register)

No commonly misidentified cell lines were used

Clinical data

Policy information about clinical studies
All manuscripts should comply with the ICMJEguidelines for publication of clinical research and a completedCONSORT checklist must be included with all submissions.

Clinical trial registration

No clinical trials were included in this study

Study protocol

No patients were involved in this study

Data collection

FRDA patient PTS and PTL clinical manifestation were provided from Dr. Caterina Mariotti (Fondazione IRCCS Istituto Neurologico "Carlo Besta")

Outcomes

No patient outcomes were included in this study
